# Making an impact: the new 2024 Medical Library Association research agenda

**DOI:** 10.5195/jmla.2025.1955

**Published:** 2025-01-14

**Authors:** Marie Ascher, Margaret A. Hoogland, Karen Heskett, Heather N. Holmes, Jonathan D. Eldredge

**Affiliations:** 1 marie.ascher@nymc.edu, Lillian Hetrick Huber Endowed Director, Phillip Capozzi, MD Library, and Assistant Professor, School of Medicine and Department of Epidemiology, New York Medical College, New York, NY; 2 Margaret.Hoogland@UToledo.edu, Associate Professor and Clinical Medical Librarian, University Libraries, The University of Toledo, Toledo, OH; 3 kheskett@ucsd.edu, Medical Librarian, University of California–San Diego Library, San Diego, CA; 4 holmesh@musc.edu, Associate Director of Libraries and Professor, Academic Affairs Faculty, The Medical University of South Carolina, SC; 5 jeldredge@salud.unm.edu, Professor, Health Sciences Library and Informatics Center and Family & Community Medicine, School of Medicine; Associate Program Director, Clinical Informatics, Graduate Medical Education; University of New Mexico, Albuquerque, New Mexico

**Keywords:** Evidence Based Practice, Research, Question Formulation, Delphi Method, Research Agenda, Consensus, Leadership, Impact, Artificial Intelligence (AI)

## Abstract

**Objective:**

This research project sought to identify those subject areas that leaders and researcher members of the Medical Library Association (MLA) determined to be of greatest importance for research investigation. It updates two previous studies conducted in 2008 and 2011.

**Methods:**

The project involved a three-step Delphi process aimed at collecting the most important and researchable questions facing the health sciences librarianship profession. First, 495 MLA leaders were asked to submit questions answerable by known research methods. Submitted questions could not exceed 50 words in length. There were 130 viable, unique questions submitted by MLA leaders. Second, the authors asked 200 eligible MLA-member researchers to select the five (5) most important and answerable questions from the list of 130 questions. Third, the same 130 MLA leaders who initially submitted questions were asked to select their top five (5) most important and answerable questions from the 36 top-ranked questions identified by the researchers.

**Results:**

The final 15 questions resulting from the three phases of the study will serve as the next priorities of the MLA Research Agenda. The authors will be facilitating the organization of teams of volunteers wishing to conduct research studies related to these identified top 15 research questions.

**Conclusion:**

The new 2024 MLA Research Agenda will enable the health information professions to allocate scarce resources toward high-yield research studies. The Agenda could be used by journal editors and annual meeting organizers to prioritize submissions for research communications. The Agenda will provide aspiring researchers with some starting points and justification for pursuing research projects on these questions.

## BACKGROUND

The Medical Library Association (MLA) research policy directs the MLA Research Caucus (formerly the Research Section) to identify “research priorities in the field” due to the limited research capacity of its membership. [1–2] MLA Research Caucus's Research Agenda Committee has implemented a research protocol for identifying these research priorities. The Research Agenda Committee in 2008 conducted a Delphi study that produced the top-ranked research questions at that time [3]. The Research Agenda Committee conducted a second Delphi study in 2011 that included refinements to the protocol [4] and published a supplemental inventory of all submitted questions [5]. The 2011 Delphi study led to a Systematic Review Project involving teams that pursued systematic or scoping reviews aimed at answering many of the top-ranked questions [6–7]. Thus, the previous MLA research agendas enabled researchers in our profession to focus their investigations on answering those specific answerable research questions that MLA leaders and researchers had identified as most important. The present study sought to improve upon past research agenda protocols while updating the research priorities in a new MLA Research Agenda.

## METHODS

### Phase One

Phase One of this Delphi method project began on September 7, 2023, with a Qualtrics survey of 495 MLA elected and appointed leaders to identify those research questions considered to be most important for the profession.

MLA leaders on the national level were defined as all elected officials, all chairs and members appointed to national level committees, and the co-editors of *JMLA*. At the caucus and chapter level, leaders were defined as all elected officers and appointed committee chairs. The authors included MLA caucus and chapter leaders to ensure the diversity of the racial, ethnic, library type, professional function, geographic location, age, life experience, and perspectives represented by these leaders. Names and email addresses were obtained from rosters on MLA Board, committee, caucus, and chapter pages, or the online MLA Member Directory. **Table 1** lists the categories of leaders with the numbers of officials filling these categories. Some of the 495 leaders served in multiple roles so the authors had to de-duplicate the leaders' names. Only those leaders whose names appeared in the MLA Member Directory were eligible to participate in Phase One of the study.

**Table 1:** MLA Leaders included in Phase One

Role	Number
National Officers	5
National Board of Directors	12
National Editors	2
National Committee Chairs	46
National Committee Members	128
Caucus Officers	235
Caucus Committee Chairs	21
Chapter Officers	92
Chapter Committee Chairs	104
**Subtotal**	**645**
Duplicates removed	147
RAC members removed	3
**Final Total**	**495**

Each leader was instructed to submit one question. The wording of the email to these leaders read: “Thank you for agreeing to participate in this brief survey on ‘What is the most important answerable research question facing the profession?’ Please enter your single sentence, most important answerable question in the following text box. Your question must be on a single topic and not exceed fifty (50) words.” Following three reminder emails sent to the same 495 MLA leaders, Phase One of the study ended on September 27, 2023.

During October 2023, one author [KH] organized the 130 viable and de-identified questions, phrases, or single-word topics submitted by the MLA leaders into broad themes. **Table 2** lists the subject themes with numbers of questions in each category with another column recording those questions also related to Artificial Intelligence (AI). The other four authors reviewed these categorized questions and discussed the subject categories among themselves and the appropriateness of specific questions in these categories. The authors reasoned that the grouping of questions into broad themes would reduce respondents' survey fatigue, making Phase Two voting easier by grouping similar questions from the long list. This is the sole manipulation of the questions and done specifically to make the voting process easier and minimize bias based on question order. Questions were reproduced in the Phase Two survey exactly how they were submitted in Phase One. Only those MLA leaders who both submitted questions and listed their names with email addresses in Phase One became eligible to participate again in Phase Three, as described later in this Methods section.

**Table 2:** Themes in Phase One Submitted Questions

Question Theme	Number of Questions	Al-related Questions
Future Casting	15	9
Information literacy/Data literacy/AI Literacy/Misinformation	12	3
Measuring the impact of librarian work	8	0
Retention/recruitment/professional development	27	2
Scholarly communication and Collections	9	0
Value of or role of librarian (broadly defined)	17	1
Value of or role of librarian, using measurement supported by data, or strategies for indicating value/role	17	0
Value of or role of librarian within technological changes	25	18

### Phase Two

Phase Two involved surveying MLA-member researchers to identify what they think are both the most important and researchable questions of those submitted in Phase One. The authors defined researchers in this study as MLA member colleagues who published peer reviewed research articles on health sciences librarianship during the years 2019 through August 2023. Additionally, as a change in protocol from previous iterations of this process, aimed at more inclusivity, MLA Research Training Institute graduates 2019–2022 and MLA research award recipients 2019–2023 were included as well. **Table 3** lists the composition of the Phase Two participants.

**Table 3:** MLA-Member Researchers included in Phase Two

Role	Number
MLA Research Paper Authors	123
MLA Research Award Winners	28
RTI Participants, not published	69
RTI Published Authors	20
**Subtotal**	**240**
3 RAC members removed	3
Duplicates Removed	37
**Deduplicated Total**	**200**

MLA member published researchers included in this study published in selected core journals. The authors defined these core journals as having peer reviewed research articles on topics that would be in-scope for MLA members:

*Evidence Based Library and Information Practice; EBLIP*

*Health Information and Libraries Journal*

*Hypothesis: Research Journal for Health Information Professionals*

*Journal of Electronic Resources for Medical Libraries; JERML*

*Journal of Hospital Librarianship*

*Journal of the Medical Library Association; JMLA*

*Medical Reference Services Quarterly.*

One investigator [MH] searched the LISTA (Library, Information Science and Technology Abstracts) database (EBSCO) for the publication years of 2019 to the present in the core journals on September 6, 2023. All articles from these six journals during this time period were reviewed for eligibility. They were found using a simple journal title search of the source field. Since only articles specifically related to health sciences librarianship were reviewed from *EBLIP*, a subject search was created for this journal title. The search strategy for these relevant articles combined searching for the journal title in the source field with the following search string: health science OR health science libraries OR health information professionals OR informationist OR informationists OR health science librarian OR solo librarian OR hospital librarian OR hospital OR health system OR health center OR health centre OR embedded librarian. In total, all of the searches combined produced 1,010 article entries that were uploaded to the Rayyantm screening platform for review.

Two authors [MH, JE] identified from those results the *research* articles published in the six core journals 2019 to August 2023. These two authors defined research as the “critical and exhaustive investigation or experimentation, having for its aim the discovery of new facts and their correct interpretation, the revision of accepted conclusions, theories, or laws in the light of newly discovered facts, or the practical application of such new or revised conclusions, theories, or laws” [8]. To be included the articles had to have an identifiable research method with measurable results for their authors to be included in this study. The authors designated over 100 “maybe” entries in their Rayyan screening platform that required a direct examination of the item to determine whether or not it fit the definition of research. The easiest items to exclude were editors' introductions, resource reviews, errata, article appendices, letters, commentaries, editorials, course descriptions, narrative reviews, or background articles. Expert consensus statements were excluded unless they contained a substantive research component. Case reports had to have a methods section, some data, and ideally a “lessons learned” section. The authors excluded surveys with fewer than 50 respondents and no measurable results. They also excluded any methods articles with fewer than 1,200 words and 10 references. History, biography, or obituary articles had to have be least 1,000 words in length and have at least 5 references.

Once the research articles in the six core journals were identified, all author names were extracted. MLA member authors were then identified using the MLA Member Directory and email addresses were recorded by two authors of the present study [HH, MA].

MLA members who received MLA Research Awards for the years 2019 through 2023 were pooled with the published researchers. Finally, those MLA member colleagues who completed the MLA Research Training Institute 2019–2023 also were added to this pool of researchers. MLA members who were identified as Leaders in Phase One were not eliminated from Phase Two. The total 200 unique researchers in this pool were invited on November 3, 2023 to participate in this second phase of the Delphi process by voting for five (5) of the 130 Phase One questions on the basis of both the (1) “importance of these questions” and (2) the “feasibility of answering these research questions.” Following three emailed reminders for the identified researchers to cast their votes, the Phase Two survey closed on February 1, 2024.

### Phase Three

In Phase Three, the 130 MLA leader participants who had submitted questions in Phase One had the final vote in determining the questions for inclusion in Research Agenda. Those participating leaders were asked by email on February 8, 2024, to vote on their top five (5) questions from the top-ranked 36 questions produced from the researchers' votes in Phase Two. Each time a potential respondent opened the survey they encountered a new randomly ordered list of 36 questions to diminish either primacy, [9] recency bias, [10] or response order bias [11–12] brought on by the sequence of questions. Phase Three ended on March 4, 2024, less than 10 months since the MLA Research Caucus Executive Board and the MLA Board of Directors had approved the study protocol.

## RESULTS

This three-phase Delphi method study produced the 15 top-ranked research questions comprising the new 2024 MLA Research Agenda that appear in **Table 4**. Phase One of the study generated 130 questions from MLA leaders.

**Table 4:** Final 15 Questions of the new MLA Research Agenda - Results of Phase Three

	Question	# of Votes
1	What is the most effective way to demonstrate the impact of librarians on health sciences research, education, and patient care?	39
2	In heavily data-driven academic medical centers and hospitals, what data should be collected and how should it be displayed and analyzed to continue to justify our value to stakeholders, including CEOs and CFOs?	30
3	What is the knowledge gap between new graduates from accredited library schools and the skills needed to work in medical libraries?	29
4	Do clinical medical librarians, by serving on rounds, provide a measurable impact on patient care (length of stay reduction, readmission reduction, etc.)?	26
5	How can we engage with diverse populations to pursue careers in health sciences librarianship?	26
6	Because so many of the people we serve don't understand what we can do or how much we can help them, how can we more effectively and actively demonstrate our value to them in a persuasive way?	24
7	Does librarian integration into health sciences instruction positively impact information seeking behaviors of health sciences trainees and professionals?	22
8	Do health sciences libraries and librarians have any measurable (statistically significant) positive impacts on consumer health, the outcomes of medical care, the productivity of biomedical researchers and the knowledge obtained by graduates of biomedical and health sciences training programs, and at what total cost?	22
9	What medical library services are most important now and what will be most important in the near future as information technology continues to rapidly evolve?	22
10	How do services provided by medical librarians contribute to the achievement of a larger institution's goals?	22
11	How will we address the fundamental changes to scholarly publishing and library budgets that are occurring with the rise of Open Science?	20
12	How can we restructure our professional organizations to meet the networking and continuing education needs of the average early career librarian via regional chapter collaborations versus a national meeting that is financially out of reach for most early and mid-career professionals?	18
13	How do we best measure long-term learning outcomes related to library-taught competencies (e.g. EPA 7) in health sciences curricula?	17
14	How will generative AI impact the health sciences librarianship profession?	17
15	How will current and future developments in artificial intelligence affect our profession - both negatively or positively?	17

Phase Two resulted in 36 questions selected by MLA-member researchers from those 130 questions as the most answerable given available research methods. Phase Three asked the participating leaders from Phase One to select their top five choices among the 36 questions that emerged from the MLA-member researchers in Phase Two. Questions 14 and 15 on Table 4 are so similar that they might be merged into one research question. The questions after Question 15 on the full list seemed repetitive. The full list of the 130 originally submitted and de-identified questions can be found in the supplemental files accompanying this article. The questions in **Table 4** have been edited to fix only punctuation and capitalization errors present in the submitted questions.

## DISCUSSION

Many of the final 15 questions are about impact. One class of questions seeks to gauge the impact of external influences upon our profession, such as the emergence of artificial intelligence, which appears twice in the final 15 questions (Q14, Q15) and new technologies (Q9 and new publishing paradigms (Q11). Another class of question asks whether health information professionals can have a measurable impact upon outcomes at their broader institutions, such as in research (Q1, Q8), education (Q1, Q7, Q8, Q13)_,_ and patient care (Q1, Q4, Q8), as well as the persistent need to demonstrate value to health care leaders (Q2, Q6, Q10). The classic Rochester Study [13] and the Detroit Study [14–15] were early attempts to answer these kinds of impact questions. Methodologically, measuring impact in broadly-defined studies makes it difficult to account for potential confounders. Lastly, two of the final 15 questions relate to the education and recruitment of new health information professionals (Q3, Q5) including the need to attract more diverse body of librarians to health sciences librarianship (Q5) and the role of professional organization in networking and continuing education (Q12). Many of the questions are similar, with some updated nuances, to those asked in 2012. All de-identified data sets for this study can be accessed in the Supplemental Files.

The next steps of the project will be to promote the new 2024 MLA Research Agenda through established MLA communication channels and to engage with colleagues who wish to join teams organized to answer one each of the 15 top-ranked research questions. The authors will facilitate the formation of these teams but will not explicitly coordinate these efforts. These new teams might wish to conduct original research, systematic reviews, or scoping reviews to address their chosen research question. These initial teams might even break into smaller teams to narrow their focus. The questions on **Table 4** inevitably will need to be refined and, in most cases, narrowed or broken down into multiple more discrete questions to be suitable for research. The new research agenda questions could be used by annual meeting organizers to recruit paper or poster presentation topics. The leading journals in our field might invite prospective researcher authors to submit manuscripts on selected top-ranked research questions. Editors might rate manuscripts on whether the research study addressed one of the top-ranked questions. The new 2024 MLA Research Agenda provides aspiring researchers with starting points and the rationale for implementing their research.

## LIMITATIONS

There are a few limitations related to this study. For one, the process was conducted during a specific period of history in the US with concerns in the larger society that might have had an outsized effect on leaders' submitted questions and the subsequent phases of votes. As only one example, after months of anxiety about possible career displacements [16–18] following OpenAI's Fall 2022 release of Chat GPT amplified in news media, it should come as no surprise that 33 artificial intelligence-related questions appeared on the initial 130 submissions. This historic artifact [19] was reduced slightly by the two subsequent rounds of voting much later in 2023 and early 2024.

Additionally, during the early portion of Phase Two an attempt to use Javascript to randomize the order of questions (in order to reduce related bias) led to a corruption of some voter output. Researchers who submitted during this phase were invited to resubmit responses. It is unknown how many of those early voters resubmitted their votes.

Lastly, some questions that remained in the study were too broadly or too vaguely stated to serve as productive questions for researchers to pursue with any known research study designs. Phase Two's inclusion of researchers culled some of these questions from further consideration. A number of the final 15 research questions will need further refinement and reframing by teams conducting original research, systematic reviews, or scoping reviews based upon a specific question. Leaders who submitted questions in Phase One were instructed to limit their single-sentence questions to no more than fifty (50) words which was less than the previous sixty (60) in the prior iteration. Future iterations of this kind of Delphi study should include additional guidelines and more detailed guidance for formulating truly answerable research questions for participants in Phase One. The investigators might want to recommend even shorter word limits to submitted questions.

## CONCLUSION

This Delphi study has produced a broad consensus statement on what subjects should be elevated in priority in the next five years. The Agenda will provide aspiring researchers with some starting points and justification for pursuing research projects on these questions. The 15 research questions in **Table 4** potentially will guide leadership and researcher collective efforts in multiple contexts to build the evidence base needed by our professional colleagues.

## Data Availability

The data associated with this project will be made available as supplemental files: the research protocol, the initial 130 questions in full, and the de-identified spreadsheets including voting tallies.

## SUPPLEMENTAL FILES

**Appendix A:** Phase 1 Survey Responses Report

**Appendix B:** Phase 2 Survey MLA Research Agenda

**Appendix C:** Phase 3 Final Tallies MLA Research Agenda 2024

**Appendix D:** Phase 3 MLA Research Agenda Delphi Study 2024
