# Supplementary material for: Making an impact: the new 2024 Medical Library Association research agenda
Source: J Med Libr Assoc. 2025 Jan 14;113(1):24–30. doi: 10.5195/jmla.2025.1955 (PMC11835027; doi:10.5195/jmla.2025.1955)
Supplement: Supplementary file 2 — Appendix B: Phase 2 Survey MLA Research Agenda [file jmla-113-1-24-s02.pdf]

Research Agenda - Round 2

Start of Block: Default Question Block

Q1 Please indicate consent below.

◯ I have read the text of the invitation email and consent to participate accordingly. (1)

End of Block: Default Question Block

Start of Block: Block 2

Q3 Thank you for agreeing to participate in this survey.

This survey requests that you review the following research questions which were submitted by MLA leaders.

Please vote for a total of five (5) of the following questions. The questions are broken into topical groups to help in your selections.

As an MLA Researcher, please place your vote based on:

1. The feasibility of answering the question with any known research study design
2. The importance of the question to the profession.

You will be required to confirm that you have only selected a total of five (5) questions across all sections before hitting submit.

Q4 Future-Casting

- [ ] As climate change increasingly poses existential threats to humanity, what steps can we take to ensure continuity of service to users while facing increasing threats to basic continued functioning (power grid failure, loss of water systems, storm damage, etc.)? (158)
- [ ] Given societal changes with misinformation, AI, publishing shifts, EMR integration, how will our profession adapt to the changes? (161)
- [ ] How AI will transform library services in the future? (162)
- [ ] How will Artificial Intelligence (AI) effect the future of libraries, librarians, and the value of a library school education? (163)
- [ ] How will artificial intelligence affect libraries and librarians? (164)
- [ ] How will current and future developments in artificial intelligence affect our profession - both negatively or positively? (165)
- [ ] How will generative AI impact the health sciences librarianship profession? (166)
- [ ] The pandemic proved that most libraries could continue providing most or all services while their physical library and/or campus was closed. What will be the long term impact of this on physical library spaces as campuses continue to maintain and add other student focused spaces offering similar amenities? (167)
- [ ] What is the future of the librarian/informationist in the health sciences? (168)
- [ ] What is the public perception of libraries & librarians in the age of AI and beyond and how does can that guide the future of our profession? (169)
- [ ] What medical library services are most important now and what will be most important in the near future as information technology continues to rapidly evolve? (170)
- [ ] what skills and qualifications will health sciences librarianship need and want to continue to contribute to improve health in the future for a diverse patient population (171)

☐ How AI (artificial intelligence) will affect the medical/ health sciences library, or even generally library, profession. (172)

☐ What effect will AI have on medical librarianship? (173)

☐ Has the 2020 pandemic shattered the profession through "wifi deserts", burnout, communication loss? (174)

Q9 Information Literacy, Data Literacy, AI Literacy, Misinformation

- ☐ How do we balance the freedom of information with the prevention of disinformation? (1)
- ☐ Effective use of artificial intelligence in designing instruction. (4)
- ☐ How can we best support and improve comprehensive, quality biomedical evidence synthesis given the enormous breadth of literature and clinician/researcher constrained resources? (5)
- ☐ How can we fight misinformation? (6)
- ☐ How can we help renew public trust in scientific information and combat mis and disinformation? (7)
- ☐ How can we take advantage of AI to better support our patrons? (8)
- ☐ How do we best measure long-term learning outcomes related to library-taught competencies (eg EPA 7) in health sciences curricula? (9)
- ☐ How do we effectively communicate the best practices use of new technologies, in this case, AI (10)
- ☐ How do we facilitate evidence-based care? (11)
- ☐ How to engage student learners and proactively connect with remote learners to promote quality literature searching and research services. (12)
- ☐ How to support scholars with information literacy and data management skills. (13)
- ☐ How do we not only keep up with but how do we contribute to solving the global crisis of health misinformation? (14)

Q10 Measuring Impact of Librarian Work

- [ ] Do clinical medical librarians, by serving on rounds, provide a measurable impact on patient care (length of stay reduction, readmission reduction, etc)? (1)
- [ ] Do health sciences libraries and librarians have any measurable (statistically significant) positive impacts on consumer health, the outcomes of medical care, the productivity of biomedical researchers and the knowledge obtained by graduates of biomedical and health sciences training programs, and at what total cost? (4)
- [ ] Does librarian integration into health sciences instruction positively impact information seeking behaviors of health sciences trainees and professionals. (5)
- [ ] How do we measure library impact on student success? (6)
- [ ] Is there demonstrable, validated research evidence of librarian-instructor impact on competency-based medical education (CBME) ? (7)
- [ ] What are effective, validated and reproducible methods for achieving desired outcomes related to librarian practice with hospital clinicians? (8)
- [ ] What is the best way to measure competency with regards to evidence-based practice skills in health sciences students? (9)
- [ ] What is the impact of a one-off library orientation on learner success? (10)

Q11 Recruitment, Retention, Professional Development

- ☐ How to engage, persist and thrive in a machine-learning, artificial intelligence informed economy, and what role will librarians play in computational knowledge management. (1)
- ☐ How can medical librarians best improve their skills to provide useful services to their users? (11)
- ☐ How can we as health sciences librarians create/contribute to a successful educational pipeline beginning in Middle or High School that leads to more BIPOC representation in the profession? (12)
- ☐ How can we continue to serve our diverse populations in the medical field, being inclusive of all their needs, while continuing to grow and diversify the profession itself- ethnicity, age, gender, skillsets, etc? (13)
- ☐ How can we engage with diverse populations to pursue careers in health sceinces librarianship? (14)
- ☐ How can we ensure the longevity and viability of existing and future medical librarians? (15)
- ☐ How can we form a stronger connection with library schools to interest students in health sciences librarianship? (16)
- ☐ How can we make our profession more inclusive? (17)
- ☐ How can we restructure our professional organizations to meet the networking and continuing education needs of the average early career librarian via regional chapter collaborations versus a national meeting that is financially out of reach for most early and mid-career professionals? (18)
- ☐ How can we retain talented information professionals with shrinking budgets and shrinking support from our institutions? (19)
- ☐ How can we support the younger generation in becoming medical librarians? (20)

- [ ] How do medical librarians adapt to AI and disinformation in their practice? (21)

- [ ] How do we best recruit, train, and support new health sciences librarians? (22)

- [ ] How do we encourage more participation in our professional associations by recent graduates and new health science librarians? (23)

- [ ] How do we make librarianship a sustainable, life-giving career in late-stage capitalism? (24)

- [ ] How do we make our profession more accessible for all people (employee and patron)? (25)

- [ ] How do we motivate quality librarians to stay in the field, when faced with a public that does not understand the role of a library and administrators that keep expecting librarians to do more with less? (26)

- [ ] How do we successfully and sustainably recruit and retain librarians of color? (27)

- [ ] How to prevent burnout? (28)

- [ ] Making sure salaries and benefits increase to retain the current workforce and inspire the next generation to become health sciences librarians (29)

- [ ] Many health sciences libraries are struggling to fill vacancies. How do we address this shortfall in qualified, interested applicants? (30)

- [ ] Our profession needs to be able to economically develop professional skills and train/re-train professionals in the latest tools/techniques/subject in pursuit of subject matter mastery and excellence faster than ever before and with limited resources. (31)

- [ ] What did you wish you learned in library school about health science librarianship? (32)

- [ ] what do we need library schools to be teaching future librarians so we have a skilled workforce to implement programs that keep academic libraries relevant (33)

- [ ] What is the knowledge gap between new graduates from accredited library schools and the skills needed to work in medical libraries? (34)

- [ ] What strategies can we use in order to keep up (both ourselves and our staff members) with the changes happening in the fields of information and health sciences education? (I don't think this is a new question, but the strategies may be.) (35)

- [ ] With the elimination of positions, how can we advance our careers? (36)

Q12 Scholarly Communications & Collections

- [ ] How do we move with the scholarly communication changing landscape? (1)
- [ ] How to provide a single gateway to all publishers. (4)
- [ ] How will we address the fundamental changes to scholarly publishing and library budgets that are occurring with the rise of Open Science? (5)
- [ ] What trainings are available and what projects have librarians worked on that relate to publication support, copyright and data management for their library patrons? (6)
- [ ] How will medical librarianship afford the costs of subscriptions and books in the next five years? (7)
- [ ] How to we prioritize information that is preserved and distributed when faced with dwindling resources and infrastructure. (8)
- [ ] My answer is sure to be colored by the kind of work I do, but I think librarianship has to decide if we are going to own the information we provide or if we are happy to simply be middle-men between ourselves and the publishers. (9)
- [ ] How do clinicians (nurses, pharmacists, etc) who do NOT have library access get full articles? (subscribe, ask a friend, Sci-hub, etc) (11)
- [ ] How will people access the information they need while costs become unsustainable? (12)

Q13 Value or Role of Librarian

- [ ] What will we do to get organized against the attack on libraries? (1)

- [ ] Among academic health sciences librarians, how does a liaison versus another model affect collaborations with non-librarian faculty? (4)

- [ ] Because so many of the people we serve don't understand what we can do or how much we can help them, how can we more effectively and actively demonstrate our value to them in a persuasive way? (5)

- [ ] Do librarians improve systematic reviews? (6)

- [ ] How do services provided by medical librarians contribute to the achievement of a larger institution's goals? (20)

- [ ] I think understanding the scope and value of what we do. (21)

- [ ] The closing of hospital libraries (22)

- [ ] The lack of awareness by those outside of the profession on the value of medical library services to healthcare. (23)

- [ ] The most important answerable question facing our profession is how to stay relevant and seen and included and consulted. (24)

- [ ] What can be done to further assist in regard to the location, enhancement, and analyzation of research results/publications to ensure healthcare professionals and the public can take the most effective and acceptable course of action when timely health-related decisions are necessary? (25)

- [ ] What is the role of the academic health sciences library in an open-access, open-science world? (26)

- [ ] Where is our assistance most sought? That is, we do -- and wish to do, and can do -- many things, but which of the services we provide are consistently most requested by our patrons? (27)

- [ ] How do we stay relevant? (28)

- [ ] How to manage all the things we have to do while operating in an always-changing context and always seeking to enhance/maintain relevance. (29)

- [ ] Value (30)

- [ ] What healthcare facilities (hospitals, nursing homes, etc.) have library access, if not, why not and what are the facilitators and barriers to having library access? Library access can mean a library of their own, participating in a consortium or other. (31)

- [ ] How to survive when we are not seen as necessary. (32)

Q14 Value or Role of Librarian - Data, Measurement, Strategies

- ☐ What are the best ways for medical librarians to advocate for an inclusive, sustainable, financially stable environment for ourselves? (1)
- ☐ What is the most effective way to demonstrate the impact of librarians on health sciences research, education, and patient care. (4)
- ☐ How can hospital librarians and libraries advocate for the importance of our profession amid an increasingly budget-conscious landscape? (5)
- ☐ How can hospital librarians and libraries advocate for the importance of our profession amid an increasingly budget-conscious landscape? (6)
- ☐ How do we continue to prove our value to administration and clinicians (7)
- ☐ How do we provide metrics on the value of librarians without de-professionalizing the work we do as "magic" or timed in 15 minute increments like a law-office billing? (8)
- ☐ How do we sustain our profession among budget cuts and technological advances that could impact how employers view our usefulness? (9)
- ☐ How to demonstrate our value to health sciences clinical practice, education, and research while correctly identifying emerging trends and practices to incorporate into our librarian practice and philosophy. (10)
- ☐ How to demonstrate the value of a fully qualified librarian in a medical setting. (11)
- ☐ In heavily data-driven academic medical centers and hospitals, what data should data be collected and how should it be displayed and analyzed to continue to justify our value to stakeholders, including CEOs and CFOs? (12)
- ☐ Is there a concrete way to show value when AI and white space created by digital collections invite people to steal our space and seek our assistance less? (13)
- ☐ What are tangible methods that can be used by a broad group of librarians (not just the usual powerful suspects) to improve professional medical practices (of non-

librarianship) and by extension daily life through informing or collaborating to improve research and reduce duplication and waste? (14)

☐ What data can be gathered that will convince Hospital Administrators (aka "The C-Suite") to appropriately fund Hospital Libraries and Librarians, whether or not said hospitals are affiliated with a large(r) academic medical center? (15)

☐ What do administrators see as the best metric of value for librarians? (16)

☐ What evidence does hospital leadership want/need to justify the value of having a librarian on staff and in a management position? (17)

☐ What is the most meaningful measure to non-librarians that shows our worth as healthcare professionals, and as having a positive effect on patient outcomes? (18)

☐ What strategies are most effective to make hospital staff and patients aware of hospital libraries and librarians? (20)

Q15 Value or Role of Librarians - Technology Changes

- [ ] How can librarians adapt and evolve to deal with increasing reliance on technology? (1)

- [ ] I think understanding the scope and value of what we do. How can medical librarians (health information professionals) adapt and leverage emerging technologies to enhance healthcare information access, delivery, and education in an increasingly digital and data-driven healthcare landscape? (4)

- [ ] How can medical librarians make their value known and ensure the continuation of the profession (in the face of AI)? (16)

- [ ] How can the work we do as medical librarians benefit from artificial intelligence tools while avoiding the inherent dangers? (17)

- [ ] How can we incorporate AI into synthesizing the abundance of literature to propel research forward faster? (18)

- [ ] How can we medical librarians use AI to improve evidence based medicine? (19)

- [ ] How ChatAI is going to impact the profession. (20)

- [ ] How do health information professionals continue to prove their value in an increasingly AI dominated world? (21)

- [ ] How do health sciences libraries communicate their role in combating the growing misinformation, climate, and ai challenges facing institutions and researchers? (22)

- [ ] How do libraries redefine their role as technology continues radically change what it takes to find information, mine or analyze text, and synthesize? (23)

- [ ] How do we demonstrate the need for health information professionals in a time of Google searches and ChatGPT? (24)

- [ ] How do we promote and preserve high-quality research and EBM during the early adoption of AI? (25)

☐ How do we remain relevant as AI becomes more stable? (26)

☐ How do we stay on the forefront of change (AI, healthcare industry changes, etc) to remain relevant and employable. (27)

☐ How will medical library professionals remain viable facing the increasing capabilities of Artificial Intelligence, including chatbots, machine learning, deep neural networks, large language models, and other emerging technologies? (28)

☐ How will we use current technologies to assist patrons in accessing information more easily and seamlessly? (29)

☐ In a post-internet and developing AI world, now more than ever, communicating that no one source has all the answers and the potential bias of any source. Ironically, a colleague suggested I ask chatGPT as I was brooding over this, it's answer was better "How can libraries adapt to the evolving information landscape and changing user needs to remain relevant and valuable in the digital age?" (30)

☐ Our profession needs to investigate the changing landscape of human/computer interactions to continue to be an intermediary between the sources of information and the humans that use it. (31)

☐ What are the current implications of emerging technologies (AI) on the perceived value and continued existence of academic librarians? (32)

☐ What are the skills and expertise medical librarians need to have to remain relevant and useful in a world with tools such as UpToDate, ChatGPT, and Google Scholar? (33)

☐ With the rapid development of artificial intelligence (AI), large language models (LLM) and machine learning (ML) technologies and their applications in many areas including our profession, how should health sciences librarians do to be better prepared for any changes to be brought over by these technologies to our profession? (34)

☐ How are we going to handle and adapt to AI (ChatGPT as an example)? (35)

☐ How best would we work with rapidly advancing AI technologies? (36)

☐ How can health sciences librarianship address "artificial intelligence" without fear or hype? (37)

☐ What practical applications does AI have for information retrieval? (38)

Q16 I have reviewed my responses and confirm that I have selected no more than five questions CUMULATIVELY across all sections above.

○ Yes (1)

Q15 **Email Address:**
Your email address is only collected for validation purposes and to prevent duplication. Once the survey closes all responses will be reviewed for validity (only five responses) and duplication (latest response will be retained). **Once the response is validated, your email address will be removed from your response.** If you selected more than five responses, you will be contacted.

____

End of Block: Block 2
