# Supplementary material for: Making an impact: the new 2024 Medical Library Association research agenda
Source: J Med Libr Assoc. 2025 Jan 14;113(1):24–30. doi: 10.5195/jmla.2025.1955 (PMC11835027; doi:10.5195/jmla.2025.1955)
Supplement: Supplementary file 4 — Appendix D: Phase 3 MLA Research Agenda Delphi Study 2024 [file jmla-113-1-24-s04.pdf]

Default Question Block

Round 3 of the MLA Research Agenda Delphi process

Please select UP TO FIVE (5) questions that you think are the most important and answerable (by research) from among those below.

Questions appear in random order.

- [ ] Because so many of the people we serve don't understand what we can do or how much we can help them, how can we more effectively and actively demonstrate our value to them in a persuasive way?
- [ ] How to demonstrate our value to health sciences clinical practice, education, and research while correctly identifying emerging trends and practices to incorporate into our librarian practice and philosophy.
- [ ] In a post-internet and developing AI world, now more than ever, communicating that no one source has all the answers and the potential bias of any source. Ironically, a colleague suggested I ask chatGPT as I was brooding over this, it's answer was better "How can libraries adapt to the evolving information landscape and changing user needs to remain relevant and valuable in the digital age?"
- [ ] How do we balance the freedom of information with the prevention of disinformation?
- [ ] How can we engage with diverse populations to pursue careers in health sciences librarianship?
- [ ] What healthcare facilities (hospitals, nursing homes, etc.) have library access, if not, why not and what are the facilitators and barriers to having library access? Library access can mean a library of their own, participating in a consortium or other.
- [ ] How can we restructure our professional organizations to meet the networking and continuing education needs of the average early career librarian via regional chapter collaborations versus a national meeting that is financially out of reach for most early and mid-career professionals?
- [ ] How do services provided by medical librarians contribute to the achievement of a larger institution's goals?
- [ ] How to support scholars with information literacy and data management skills.
- [ ] Among academic health sciences librarians, how does a liaison versus another model affect collaborations with non-librarian faculty?

- [ ] Does librarian integration into health sciences instruction positively impact information seeking behaviors of health sciences trainees and professionals.
- [ ] How will we address the fundamental changes to scholarly publishing and library budgets that are occurring with the rise of Open Science?
- [ ] What is the knowledge gap between new graduates from accredited library schools and the skills needed to work in medical libraries?
- [ ] As climate change increasingly poses existential threats to humanity, what steps can we take to ensure continuity of service to users while facing increasing threats to basic continued functioning (power grid failure, loss of water systems, storm damage, etc.)?
- [ ] Making sure salaries and benefits increase to retain the current workforce and inspire the next generation to become health sciences librarians
- [ ] What skills and qualifications will health sciences librarianship need and want to continue to contribute to improve health in the future for a diverse patient population
- [ ] How can we best support and improve comprehensive, quality biomedical evidence synthesis given the enormous breadth of literature and clinician/researcher constrained resources?
- [ ] How can we fight misinformation?
- [ ] What medical library services are most important now and what will be most important in the near future as information technology continues to rapidly evolve?
- [ ] What is the most effective way to demonstrate the impact of librarians on health sciences research, education, and patient care.
- [ ] Do health sciences libraries and librarians have any measurable (statistically significant) positive impacts on consumer health, the outcomes of medical care, the productivity of biomedical researchers and the knowledge obtained by graduates of biomedical and health sciences training programs, and at what total cost?
- [ ] How do we measure library impact on student success?
- [ ] Is there demonstrable, validated research evidence of librarian-instructor impact on competency-based medical education (CBME) ?
- [ ] How do we not only keep up with but how do we contribute to solving the global crisis of health misinformation?
- [ ] The pandemic proved that most libraries could continue providing most or all services while their physical library and/or campus was closed. What will be the long term impact of this on physical library spaces as campuses continue to maintain and add other student focused spaces offering similar amenities?
- [ ] What is the public perception of libraries & librarians in the age of AI and beyond and how does can that guide the future of our profession?
- [ ] Given societal changes with misinformation, AI, publishing shifts, EMR integration, how will our profession adapt to the changes?

- ☐ What is the best way to measure competency with regards to evidence-based practice skills in health sciences students?
- ☐ How will current and future developments in artificial intelligence affect our profession - both negatively or positively?
- ☐ What is the future of the librarian/informationist in the health sciences?
- ☐ How do clinicians (nurses, pharmacists, etc) who do NOT have library access get full articles? (subscribe, ask a friend, Sci-hub, etc)
- ☐ In heavily data-driven academic medical centers and hospitals, what data should data be collected and how should it be displayed and analyzed to continue to justify our value to stakeholders, including CEOs and CFOs?
- ☐ How can we help renew public trust in scientific information and combat mis and disinformation?
- ☐ How do we best measure long-term learning outcomes related to library-taught competencies (eg EPA 7) in health sciences curricula?
- ☐ Do clinical medical librarians, by serving on rounds, provide a measurable impact on patient care (length of stay reduction, readmission reduction, etc)?
- ☐ How will generative AI impact the health sciences librarianship profession?

Powered by Qualtrics
